# Deep Learning-Based Multi-Omics Integration Robustly Predicts Relapse in Prostate Cancer

**DOI:** 10.3389/fonc.2022.893424

**Published:** 2022-06-23

**Authors:** Ziwei Wei, Dunsheng Han, Cong Zhang, Shiyu Wang, Jinke Liu, Fan Chao, Zhenyu Song, Gang Chen

**Affiliations:** ^1^ Department of Urology, Jinshan Hospital, Fudan University, Shanghai, China; ^2^ Department of Urology, Zhongshan Hospital, Fudan University (Xiamen Branch), Xiamen, China; ^3^ Ovarian Cancer Program, Department of Gynecologic Oncology, Zhongshan Hospital, Fudan University, Shanghai, China

**Keywords:** prostate cancer, relapse prediction, multi-omics, autoencoder, deep learning, H2O package

## Abstract

**Objective:**

Post-operative biochemical relapse (BCR) continues to occur in a significant percentage of patients with localized prostate cancer (PCa). Current stratification methods are not adequate to identify high-risk patients. The present study exploits the ability of deep learning (DL) algorithms using the H2O package to combine multi-omics data to resolve this problem.

**Methods:**

Five-omics data from 417 PCa patients from The Cancer Genome Atlas (TCGA) were used to construct the DL-based, relapse-sensitive model. Among them, 265 (63.5%) individuals experienced BCR. Five additional independent validation sets were applied to assess its predictive robustness. Bioinformatics analyses of two relapse-associated subgroups were then performed for identification of differentially expressed genes (DEGs), enriched pathway analysis, copy number analysis and immune cell infiltration analysis.

**Results:**

The DL-based model, with a significant difference (P = 6e-9) between two subgroups and good concordance index (C-index = 0.767), were proven to be robust by external validation. 1530 DEGs including 678 up- and 852 down-regulated genes were identified in the high-risk subgroup S2 compared with the low-risk subgroup S1. Enrichment analyses found five hallmark gene sets were up-regulated while 13 were down-regulated. Then, we found that DNA damage repair pathways were significantly enriched in the S2 subgroup. CNV analysis showed that 30.18% of genes were significantly up-regulated and gene amplification on chromosomes 7 and 8 was significantly elevated in the S2 subgroup. Moreover, enrichment analysis revealed that some DEGs and pathways were associated with immunity. Three tumor-infiltrating immune cell (TIIC) groups with a higher proportion in the S2 subgroup (p = 1e-05, p = 8.7e-06, p = 0.00014) and one TIIC group with a higher proportion in the S1 subgroup (P = 1.3e-06) were identified.

**Conclusion:**

We developed a novel, robust classification for understanding PCa relapse. This study validated the effectiveness of deep learning technique in prognosis prediction, and the method may benefit patients and prevent relapse by improving early detection and advancing early intervention.

## Introduction

Data accumulation is increasing exponentially with the development and application of advanced technologies such as chips and sequencing in the biomedical field. Combined with state-of-the-art algorithms, it is revealing strong biological associations in the pathomechanism of various cancers (1, 2). Before this new era, cancer studies concerning single-dimensional data could only obtain limited information, but multi-omics data integration approaches can now address important biological questions. Multi-omics data integration techniques have been widely applied for identifying subtypes, and multiple studies have revealed that the deep learning (DL) method may be effective for transducing multi-omics data to construct more accurate prognosis models (3, 4).

Prostate cancer (PCa) is one of the most common malignancies in elderly men, accounting for 26% of all cancers and 11% of estimated cancer death in males in 2021 (5). After the PCa patients received either radical prostatectomy (RP) or external beam radiotherapy, 27−53% of patients experienced biochemical recurrence (BCR) (6). Combined with surgical margin status, clinically applied prognostic factors such as prostate specific antigen (PSA) value, tumor-node-metastasis (TNM) status and Gleason score can help assess the risk of relapse after RP (7). However, these parameters lack predictive accuracy. As we all know, the best medical decisions should be made according to the patients’ specific situations. Relapse is indeed a very significant part of it, and prediction represents a major challenge (8). New methods to discover relapse-sensitive subtypes are much needed, and a more accurate risk-stratification tool improve the allocation of medical resources (9).

In recent years, several studies have identified PCa molecular subtypes (10–14). Huang et al. (10) generated a set of long non-coding RNAs (lncRNAs) to predict BCR-free survival of PCa using The Cancer Genome Atlas (TCGA; https://www.cancer.gov/) dataset, a large and detailed database including omics data for more than 30 cancer types. The results showed that this four-lncRNA model was more precise than the American Joint Committee on Cancer T stage and Gleason score, although differences were not significant. Chu et al. (11) used a random forest-based variable hunting approach to select eight messenger RNA (mRNA), and developed a risk score staging system. Importantly, this eight-gene model was further validated by another independent dataset. Wang et al. (13) integrated mRNA, microRNA (miRNA), and methylation data, selected TELO2, ZMYND19, miR-143, miR-378a, cg00687383, and cg02318866 for model construction, and reported a high concordance index (C-index = 0.713).

Genomics, epigenomics, transcriptomics, and other omics approaches can broadly be defined as systematic methods for collecting multifarious biological data, and these techniques can reveal the heterogeneity of tumors and provide new types of molecular classification. The H2O Deep Learning Estimator has not been applied to PCa relapse prediction, and meanwhile one or a few omics layers have been considered in previous studies, with a small number of biomarkers. To more comprehensively mine multi-omics data, we herein developed a robust relapse risk-stratification model for PCa based on up to five-omics data using the H2O Deep Learning Estimator, consisting of mRNA, miRNA, DNA methylation, copy number variations (CNVs), and lncRNA. Five external validation sets were employed to evaluate its robustness, which was lacking for previous predictive models. Furthermore, detailed bioinformatic analysis was performed from multiple perspectives. We evaluated differentially expressed genes (DEGs), critical signaling pathways, CNVs, and tumor-infiltrating immune cell (TIIC) groups associated with PCa relapse.

## Materials and Methods

### Data Acquisition and Study Design

**Figure 1:**
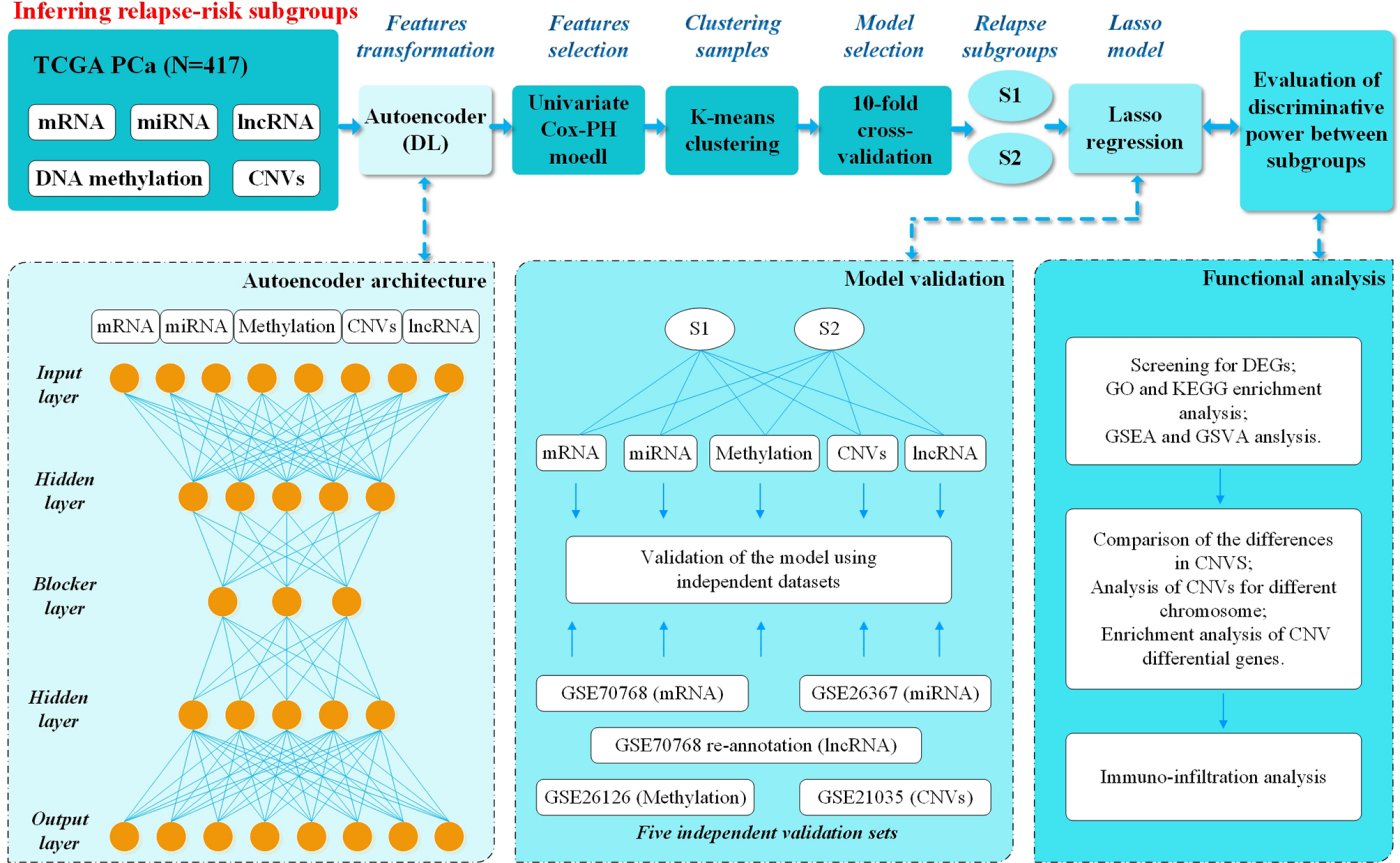
Overall workflow. Firstly, mRNA, miRNA, DNA methylation, CNVs, and lncRNA deep features from the TCGA PCa cohort were stacked up as input features for the autoencoder, a deep-learning method. Then each of the new and transformed features in the bottleneck layer of the autoencoder were subjected to univariate Cox-PH models to select the features associated with relapse. Then K-mean clustering was applied to relapse-associated deep features and 10-fold cross-validation was applied to analyze the C-index for different clusters and relapse in 8 DL models. The best model (model_3) with better discriminative ability was finally selected using Kaplan-Meier plotter between two models with the highest C-index. Then the Lasso method was used to filter out the relapse-associated feature labels, according to model_3 subgroups, from the database of TCGA, including mRNA, miRNA, DNA methylation, CNVs and lncRNA. The Lasso model was constructed, and five external validation sets from GEO were used to evaluate its prediction ability. Last but not least, functional analysis was performed to understand the different characteristics between two relapse-associated subgroups.

Multi-omics PCa data from TCGA, including mRNA, miRNA, DNA methylation, CNVs and lncRNA, were subjected to dimensionality reduction analysis to extract associated genes using the H2O package in R (v3.6.0) (15), an open-source machine learning platform that supports the most widely used machine learning models and advanced models, such as DL and so forth. Multi-omics datasets were obtained from the TCGA data portal. CNV values were generated by GISTIC 2.0, and processing of methylation data was conducted as previously described (3, 9). Hyperparameter optimization was performed by grid search, and DL models were then built. Five additional validation sets were applied to evaluate the predictive robustness of the best-performing model. The study workflow is shown in **Figure 1**.

An autoencoder with three hidden layers (n = 50, 100, 150, 200, 250, 300, 400, 500) was implemented, for which the bottleneck layer was used to discover new labels from the multi-omics data, with α_a_ = 0.0001 and α_w_ = 0.001. As reported, selection parameters to train the autoencoder were ‘TanhWithDropout’ as the activation function, ‘log loss’ as the objective function, and ‘10 epochs and 50% dropout’ as the gradient descent algorithm (3).In detail taking the input *x* = (*x*_1_, ..., *x_n_*), the output of *x* ^′^, for a given *i* the formula is *x* ^′^ = *tanh*(*W*_*i*_ *x*+*b*_*i*_) where *W_i_* is the weight matrix of size *x*×*x* ^′^. For *k* layer in the autoencoder, the formula is *x* ^′^ *F*_1→*k*_(*x*)=*f*_1_°...°*f*_*k*−1_°*f*_*k*_(*x*) $logloss\left(x,x^{\prime }\right)=\sum_{k-1}^{d}\left(x_{k}log\left(x_{k}^{'}\right)+\left(1-k\right)log\left(1-x_{k}^{'}\right)\right)$ was applied to measure the error between the input and output. To control overfitting, $L\left(x,x^{\prime }\right)=logloss\left(x,x^{\prime }\right)+\sum_{i=1}^{k}\left(\alpha_{w}{\left|\left|W_{i}\right|\right|}_{1}+\alpha_{a}{\left|\left|F_{1\to i}\left(x\right)\right|\right|}_{2}^{2}\right)$ . Finally, eight DL models were built based on different hidden layers.

### Robustness Assessment and Model Selection

We extracted deep features from eight DL models, and features related to relapse were screened out by univariate Cox proportional hazards (Cox-PH). Next, we used the K-means clustering algorithm to cluster the samples. Different clusters and relapse C-index were evaluated by 10-fold cross-validation.

Then, patients were divided into two subgroups based on relapse-associated deep features according to the models with good C-index scores, and KM plot was used to analyze the relapse level. Finally, the most suitable model was chosen for subsequent validation.

### Lasso Model Building and DL Model External Validation

The Lasso method was used to filter out relapse-associated feature labels from the TCGA database depending on the chosen model, including mRNA, miRNA, DNA methylation, CNVs and lncRNA. Five additional validation sets from the GEO database, an international public functional genomics data repository, were applied to assess the predictive effectiveness of this DL-based relapse prediction model (i.e., GSE70768 for mRNA, GSE70768 re-annotation for lncRNA, GSE26367 for miRNA, GSE26126 for DNA methylation, and GSE21035 for CNVs). Log-rank *p*-value and C-index were applied for performance evaluation.

### Bioinformatics Analysis

The characteristics of two relapse-associated subgroups of TCGA PCa samples were explored through multiple bioinformatics analysis.

#### Identification of DEGs

To identify DEGs between the two subgroups, differential gene expression analysis was performed for each omics data type. The DESeq2 R package (16) was used to filter DEGs between the two subgroups (absolute (log_2_ fold change >0.585 and adjusted *p*-value <0.01). The *lumi* and *limma* R packages were applied for processing DNA methylation (17–19), and filtering was defined as averaged *M* value differences >1.

#### Enriched Pathway Analysis

The clusterProfiler R package was used to perform the GO and KEGG enrichment analyses (20). Up- and down-regulated genes and pathways were separately assessed. The GO and KEGG enrichment analysis results were visualized as bubble plots. GSEA was also performed using the clusterProfiler package. Hallmark gene sets c2.cp.kegg.v6.2.symbols.gmt, c2.cgn.v6.2.symbols.gmt, c5.all.v6.2.symbols.gmt and c6.all.v6.2.symbols.gmt were downloaded from the MSigDB molecular signatures database (http://software.broadinstitute.org/gsea/msigdb). GSVA package was then implemented, and the single sample GSEA method was used for hallmark gene sets to further calculate the GSVA scores of each gene set for each sample.

#### Copy Number Analysis

Firstly, Wilcoxon’s signed-rank test was used to compare differentially expressed CNVs between two subgroups. Secondly, the Heatmap function in R was used to present copy number heatmaps. Thirdly, copy number frequency and gistic score in different chromosomes was generated by GISTIC 2.0. Finally, GO enrichment analysis was carried out for CNV differential genes, proportional Venn diagrams were generated with a Venn diagram plotter, and functional enrichment analysis was then separately performed for up- and down-regulated genes.

#### Immune Cell Infiltration Analysis

The CIBERSORT algorithm was used to calculate the proportion of infiltrating immune cell subsets, and 22 types of immune cells were detected in these PCa samples. Cells with statistically significant differences were screened and analyzed by Wilcoxon’s signed-rank test, with a threshold of *p <*0.05.

## Results

### Identification of Two Differential Relapse Subgroups in TCGA PCa Samples

A total of 417 tumor samples were obtained from the TCGA PCa project, which included five-omics data (mRNA, miRNA, DNA methylation, CNVs and lncRNA). In our study population, all patients underwent RP due to PCa, and 265 (63.5%) experienced BCR while 152 (36.5%) did not. As mentioned in the Materials and Methods, we subsequently performed preprocessing of this data. The autoencoder architecture or DL framework was applied (**Figure 1**), which stacked these five-omics features together.

Eight DL models were constructed based on the different hidden layers. Univariate Cox-PH regression on each of the deep features as then performed to verify significance (Wald test *p*-value <0.05) associated with relapse. We used K-means for clustering analysis and 10-fold cross-validation (CV) to calculate C-index for different clusters related to relapse. The results showed that all eight DL framework models generated a good C-index value (>0.64), and this value was >0.75 for model_3 and model_8 (**Table 1**).

**Table 1:** Characteristics of eight DL models.

ae_models	hidden	epochs	activation	hidden_dropout_ratios	l1	l2	model_ids	rmse	deep_features	mean c-index/SD
model_1	[500, 50, 500]	10	TanhWithDropout	[0.5, 0.5, 0.5]	0.001	1.00E-05	ae_grid3_model_12	0.400865	8	0.647/0.034
model_2	[500, 100, 500]	10	TanhWithDropout	[0.5, 0.5, 0.5]	1.00E-05	0.1	ae_grid3_model_16	0.400872	12	0.649/0.026
model_3	[500, 150, 500]	10	TanhWithDropout	[0.5, 0.5, 0.5]	0.001	0	ae_grid3_model_15	0.400877	21	0.767/0.020
model_4	[500, 200, 500]	10	TanhWithDropout	[0.5, 0.5, 0.5]	0.1	1.00E-04	ae_grid3_model_3	0.400877	35	0.706/0.055
model_5	[500, 250, 500]	10	TanhWithDropout	[0.5, 0.5, 0.5]	0.1	0	ae_grid4_model_10	0.400881	42	0.749/0.027
model_6	[500, 300, 500]	10	TanhWithDropout	[0.5, 0.5, 0.5]	0.01	0	ae_grid4_model_4	0.400886	57	0.668/0.021
model_7	[500, 400, 500]	10	TanhWithDropout	[0.5, 0.5, 0.5]	1.00E-05	0.001	ae_grid4_model_1	0.400889	60	0.723/0.020
model_8	[500, 500, 500]	10	TanhWithDropout	[0.5, 0.5, 0.5]	0	0	ae_grid4_model_14	0.40089	96	0.775/0.016

**Figure 2:**
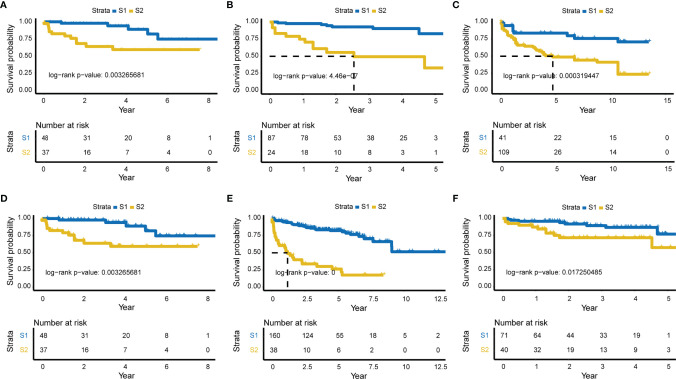
Significant survival differences for model _3 and five external validation sets. Relapse-related deep-features of model_3 were used for subgrouping, and KM plot was used to show the difference in relapse levels between the two subgroups. The Lasso model constructed according to model_3 was validated in each of the five external validation sets. **(A)** KM plot of model_3 (log-rank *P*-value = 6e-09, the time of half relapse is about 3.5 years). **(B)** GSE70768 validation set (mRNA, Number of samples = 111, log-rank *p*-value = 4.46e-07). **(C)** GSE26367 validation set (miRNA, N = 149, log-rank *P*-value = 0.000319447). **(D)** GSE26126 validation set (DNA methylation, N = 85, log-rank *P*-value = 0.003265681). **(E)** GSE21035 validation set (CNVs, N = 198, log-rank *P*-value = 0), and **(F)** Re-annotated GSE70768 validation set (mRNA, N = 111, log-rank *P*-value = 0.017250485).

Subgrouping procedures were separately employed using the relapse-related deep features obtained from model_3 and model_8. Relapse differences between subgroups were then evaluated by Kaplan-Meier plotter (KM plot). Two subgroups of model_3 revealed more significant differences (log-rank *p*-value = 6e-09), with a time to relapse ~3.5 years for half of patients (**Figure 2A**, **Supplementary Figure 1**).

### Evaluation of Relapse in Five Independent Validation Sets

Characteristic labels were selected and a Lasso model was constructed, with 43 mRNAs, 22 miRNAs, 24 lncRNAs, 30 methylation genes, and 72 CNV genes (**Supplementary Table 1**). Five independent validation sets from the Gene Expression Omnibus (GEO; http://www.ncbi.nlm.nih.gov/geo/) database were then applied to demonstrate the predictive classification robustness of the model for PCa relapse outcomes. Each of the validation sets represented mRNA, miRNA, DNA methylation, CNVs, or lncRNA, respectively (**Figures 2B–F**). The GSE70768 dataset was a mRNA validation set with 111 patients, which had a log-rank *p*-value of 4.46e-07 between the two PCa relapse-associated subgroups (low-risk S1 vs. high-risk S2; **Figure 2B**). The GSE26367 miRNA validation set consisted of 150 samples with a log-rank *p*-value of 0.000319447 between S1 and S2 (**Figure 2C**). The GSE26126 DNA methylation validation set included 85 samples, and the two subgroups yielded a log-rank *p*-value of 0.003265681 (**Figure 2D**). The GSE21035 CNVs validation set with 198 patients had an extremely low log-rank *p*-value of 0 between the two subgroups (**Figure 2E**). Finally, the GSE70768 re-annotated lncRNA validation set had a log-rank *p*-value of 0.017250485 between S1 and S2 (**Figure 2F**).

### Analysis of DEGs in Relapse Subgroups

**Figure 3:**
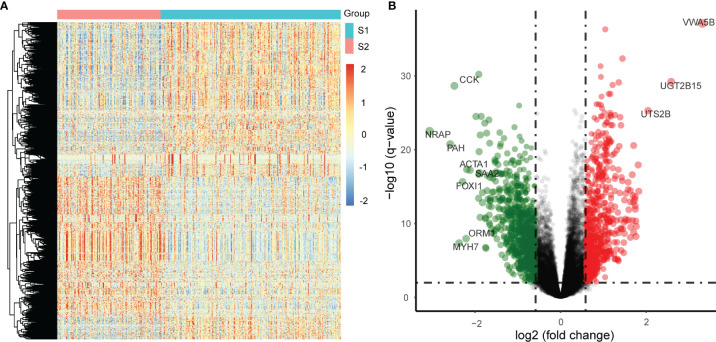
Differentially expressed genes (DEGs) in the two subgroups from the TCGA PCa samples. **(A)** Differential expression: S2 vs S1, S1: a low relapse-risk subgroup of PCa, S2: a high relapse-risk subgroup of PCa. **(B)** Volcano plot of DEGs.

DEGs between the two identified subgroups were identified by the DESeq2 package. After applying adjusted *p*-value <0.01 and absolute fold change >1.5 as cut-off criteria, we obtained 1530 DEGs including 678 up-regulated and 852 down-regulated genes in the S2 subgroup (the high relapse risk subcluster) compared to S1 (the low relapse risk subcluster). Gene expression profile comparisons of these 1530 genes after normalisation is shown in **Figure 3A**, and the results are presented as a volcano plot (**Figure 3B**). The three most significantly up-regulated genes in the S2 subgroup, von Willebrand factor a domain-containing 5B1 (VWA5B1), uridine 5’-diphosphate glucuronosyltransferase 2B15 (UGT2B15), and urotensin II-related peptide (URP, also called UTS2B), all with log2[fold change] >2 and -log10[*q*-value] >2, are related to genetic polymorphisms (21–23). In addition, down-regulated genes such as CCK, NRAP and PAH (log2[fold change] <-2 and -log10[*q*-value] >2) were also noted.

**Figure 4:**
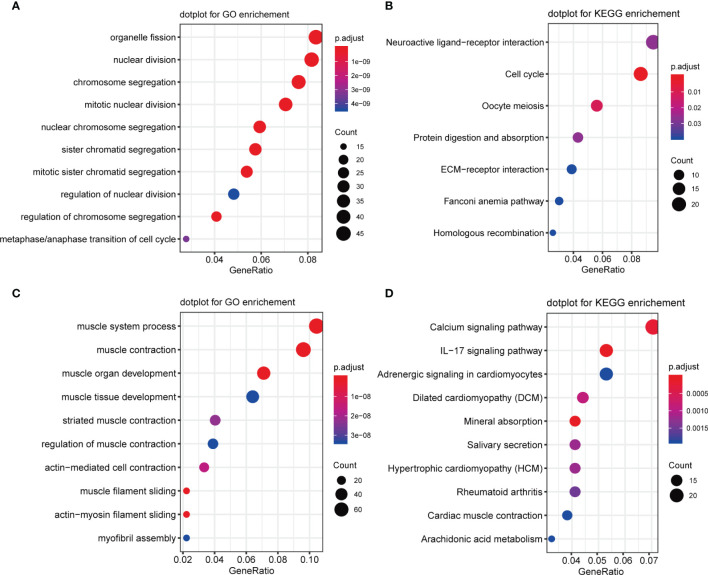
GO and KEGG enrichment of upregulated and downregulated genes. **(A)** GO enrichment analysis of upregulated genes. **(B)** KEGG enrichment analysis of upregulated genes. **(C)** GO enrichment analysis of downregulated genes. **(D)** KEGG enrichment analysis of downregulated genes.

Next, Gene Ontology (GO) and Kyoto Encyclopedia of Genes and Genomes (KEGG) pathway analyses were performed on DEGs which were significantly up-regulated (fold change >2 and *p <*0.05) or down-regulated (fold change <-2 and *p <*0.05). GO analysis results for up-regulated genes were enriched with cancer-related cell proliferation terms such as organelle fission, nuclear division, chromosome segregation, mitotic nuclear division, nuclear chromosome segregation, metaphase/anaphase transition of the cell cycle, and others (**Figure 4A**), KEGG analysis results showed that up-regulated genes were also involved in the cell cycle, and some other pathways including neuroactive ligand-receptor interaction, cell cycle, oocyte meiosis, protein digestion, and absorption were also highly enriched (**Figure 4B**). GO analysis revealed that these down-regulated genes were enriched in many muscle-related biological process terms including muscle system process, muscle organ/tissue development, actin-mediated cell contraction, actin-myosin filament sliding, and myofibril assembly (**Figure 4C**). KEGG analysis showed that DEGs were enriched in calcium signaling, IL-17 signaling, adrenergic signaling in cardiomyocytes, dilated cardiomyopathy (DBM), mineral absorption, salivary secretion, and others (**Figure 4D**).

**Figure 5:**
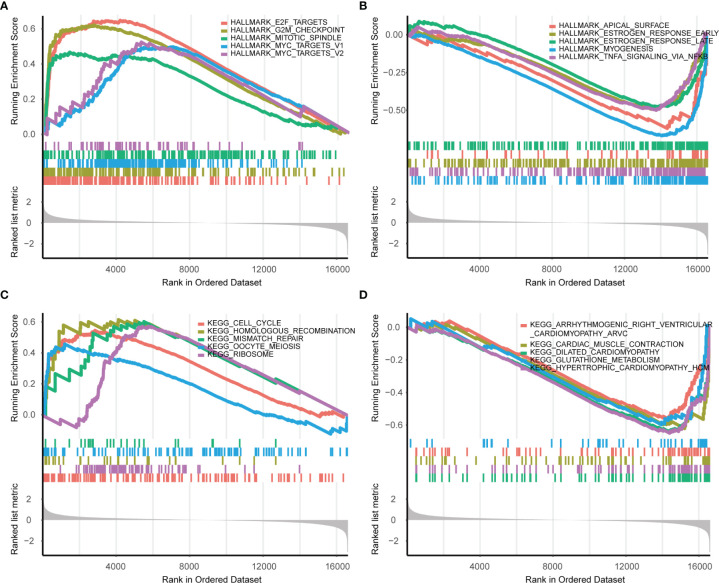
GSEA enrichment analysis in Hallmarks and KEGG (S2 vs S1). **(A)** The top five upregulated hallmarks. **(B)** The top five downregulated hallmarks. **(C)** The top five upregulated pathways in KEGG. **(D)** The top five downregulated pathways in KEGG.

Next, Gene set enrichment analysis (GSEA) was performed and the results indicated several malignant hallmarks and pathways of cancer, of which the top five up-regulated hallmarks were E2F targets, G2/M checkpoint, mitotic spindle, myc targets v1 and myc targets v2 (**Figure 5A**), and the top five down-regulated hallmarks were apical surface, estrogen response early, estrogen response late, myogenesis, and TNFA signaling *via* NF-kb (**Figure 5B**). Additionally, several malignant KEGG pathways of cancer were identified, among which the top five up-regulated pathways were cell cycle, homologous recombination, mismatch repair, oocyte meiosis and ribosome (**Figure 5C**), and the top five down-regulated pathways were arrhythmogenic right ventricular cardiomyopathy, cardiac muscle contraction, dilated cardiomyopathy, glutathione metabolism and hypertrophic cardiomyopathy hcm (**Figure 5D**).

**Figure 6:**
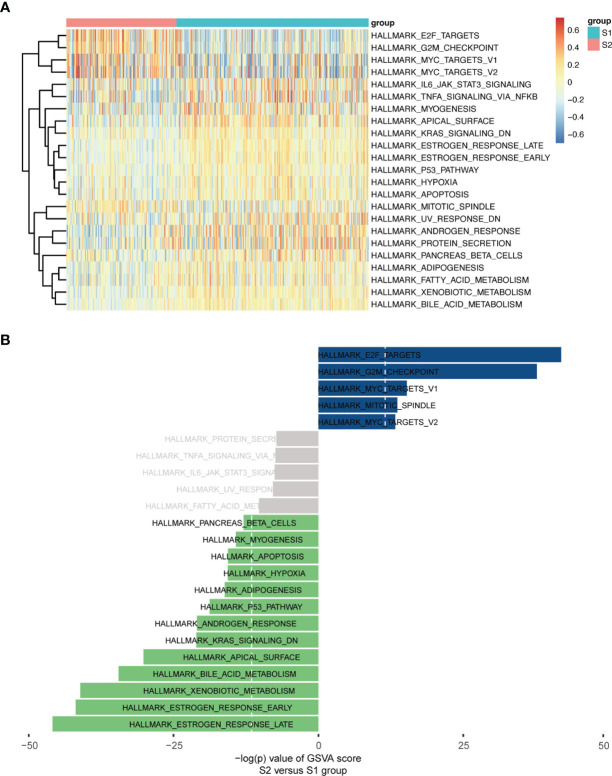
GSVA enrichment analysis in Hallmarks. **(A)** Heatmap plot. **(B)** Bar Chart (-log(p) value of GSVA score were used, S2 vs S1).

Moreover, a heatmap plot was used to present the different expression levels of hallmark gene sets (**Figure 6A**), and a bar chart further showed 23 differentially expressed hallmark gene sets based on -log(p) value of the gene set variation analysis (GSVA) score order (**Figure 6B**). Five hallmark gene sets were up-regulated (-log(p) value of GSVA score >10), and 13 hallmark gene sets were down-regulated (-log(p) value of GSVA score <-10).

### CNVs Analysis

**Figure 7:**
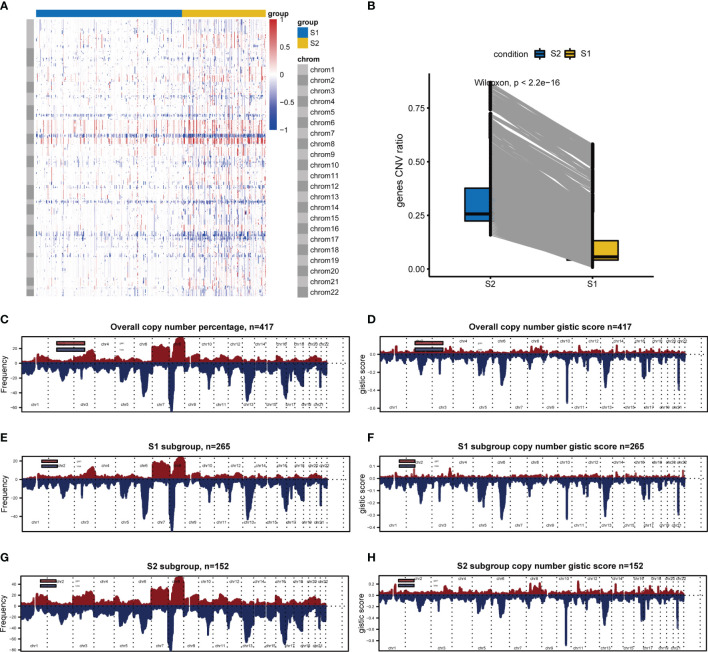
CNVs difference analysis between S1 and S2. **(A)** CNVs difference analysis by Wilcoxon. **(B)** Hierarchical clustering. Red indicates amplification, whereas blue indicates deletion. **(C–H)** Chromosomal distribution of copy number by GISTIC. Red indicates amplification, whereas blue indicates deletion.

Since analysis of functional difference outcomes showed that DNA damage repair pathways such as homologous recombination and mismatch repair were significantly enriched in the S2 subgroup, we compared differences in CNVs between the two subgroups. The results showed that 30.18% (7844/25988) of genes in the S2 subgroup were significantly up-regulated, and no significant differences were found in other genes between the two subgroups (*p <*2.2e-16; **Figure 7A**). Further analysis was performed on the gene copy number for different chromosomes, with 265 samples in the S1 subgroup and 152 samples in S2 subgroup, and the results showed that gene amplification on chromosomes 7 and 8 in the S2 subgroup was significantly greater than that in the S1 subgroup (**Figure 7B–H)**.

**Figure 8:**
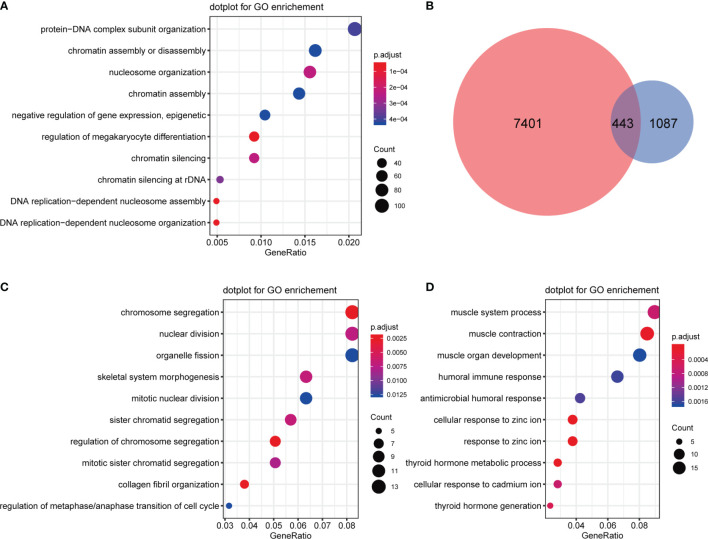
Functional analysis of CNV differential genes. **(A)** GO enrichment analysis of CNV differential genes. **(B)** Venn diagrams of CNV differential genes and expression differential genes. **(C)** GO enrichment analysis of upregulated CNV genes. **(D)** GO enrichment analysis of downregulated CNV genes.

GO analysis was applied for CNV differential genes, and the results showed that protein-DNA complex subunit organization, chromatin assembly, disassembly and silencing, nucleosome organization, negative regulation of gene expression (epigenetic), and DNA replication-dependent nucleosome assembly and organization were enriched (**Figure 8A**). Regarding overlapping CNV differential genes and expression differential genes, 443 gene expression levels were altered, of which 190 were up-regulated and 253 were down-regulated (**Figure 8B**). Subsequent GO analysis of up-regulated CNV genes revealed enrichment in chromosome segregation, nuclear division, organelle fission, skeletal system morphogenesis, mitotic nuclear division, and others (**Figure 8C**), and down-regulated CNV genes were enriched in muscle system process, antimicrobial humoral response, cellular response to zinc ion, and thyroid hormone metabolic process terms. Interestingly, a humoral immune response was also involved (**Figure 8D**).

### Analysis of Tumor-Infiltrating Immune Cells

**Figure 9:**
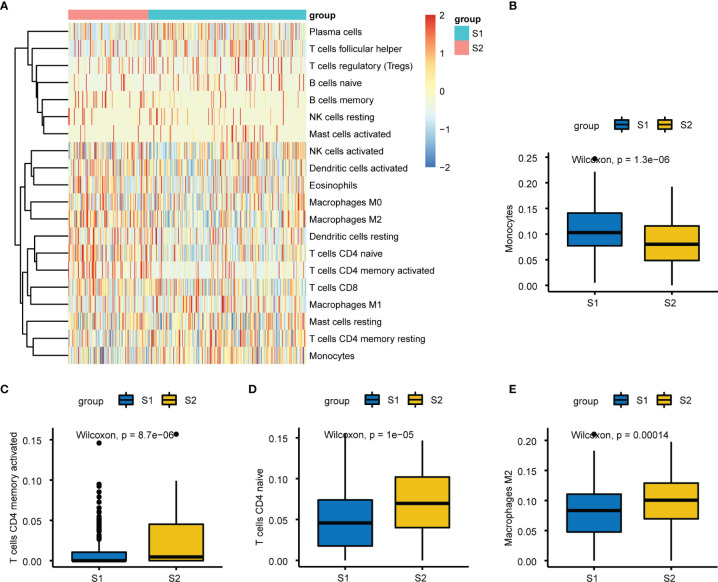
Immuno-infiltration analysis between S1 and S2. **(A)** Heatmap plot of the 22 tumors infiltrating immune cells (TIICs) in two groups. **(B–E)** Relative proportion of the differential four types of TIICs between two subgroups, respectively.

We calculated and displayed 22 TIICs per sample from TCGA analysis for the two subgroups with significantly different relapse-risk subgroups. The heatmap shows the relative levels of TIICs between the two sample subgroups (**Figure 9A**). Finally, four types of TIICs, namely CD4 naïve T cells, CD4 memory-activated T cells, monocytes, and M2 macrophages, differed significantly between the two subgroups (**Table 2**, **Figure 9B–E**). Among them, M2 macrophages, CD4 naïve T cells and CD4 memory-activated T cells were more abundant in the S2 subgroup (*p* = 0.00014, *p* = 1e-05, *p* = 8.7e-06), while monocytes were more abundant in the S1 subgroup (*p* = 1.3e-06).

**Table 2:** Characteristics of four significantly different tumor-infiltrating immune cells.

Cell type	S1 subgroup	S2 subgroup	*p*-value	adj *p*-value
T cells CD4 naive	0.03706419	0.061121026	1.375140e-05	0.0002475252
T cells CD4 memory active	0.00000000	0.002554248	8.680098e-06	0.0001649219
Monocytes	0.08214986	0.061738947	8.389214e-04	0.0142616642
Macrophages M2	0.06320131	0.082005050	5.701414e-06	0.0001140283

## Discussion

Although patients with localized PCa undergoing RP may have favorable oncological results, the incidence of BCR can reportedly be more than one-fourth (24). Thus, it is clinically important for urologists to distinguish patients at high risk of relapse from those with low risk to initiate early salvage treatment, while for those with a low risk of relapse, treatment can be deferred. Although there are some studies on relapse prognostication of PCa patients, incorporating multi-omics data to identify subgroups has rarely been reported (25, 26). In addition, most published PCa subgroup models have either no or very few independent validation sets, hence the predictive values of these identified subgroups are not very satisfactory. Thus, new practical procedures in which a predictive model could feed back the relapse outcome of PCa patients directly are needed.

DL has emerged as a versatile approach for predicting complex biological phenomena (27). Previous DL-based models of PCa were mainly applied in preclinical discovery, Gleason grading, and tumor metastasis (28–31). Baek et al. (32) proposed two biological features based on mRNA, miRNA, and methylation datasets to predict high-risk pancreatic adenocarcinoma, which achieved good performance with C-index ~0.8 for both disease-free survival and overall survival. In terms of liver cancer, a previous DL-based three-omics (mRNA, miRNA, and methylation) integration robustly predicted survival, with C-index = 0.68. Furthermore, another support vector machine model using bidirectional deep neural networks integrating DNA methylation and mRNA expression data could also cluster samples into two survival subgroups (3, 33). Herein, we identified differences in relapse risk between two subgroups of PCa patients using five-omics data, and the model performed well, with C-index >0.75 and log-rank *p*-value = 6e-9 between the two relapse subgroups. To our knowledge, this is the first application of a DL framework for integrating five different datatypes to predict relapse of PCa, and it achieved the best C-index reported to date and included sufficient external confirmation cohorts.

The open-source H2O platform is a powerful tool that can automatically select DL strategies and parameter settings to predict biological differences (34). Furthermore, automated machine learning has great value for many areas of medicine, especially in cancer diagnosis and judging prognosis (35–37). In the present study, we established eight DL-based predictive models for PCa relapse according to different hidden layers using H2O. The results showed that the final selected model (model_3) was robust and might be superior to others, including previous prediction models, for several levels. Because of its ability to achieve more accurate outcomes and its great universality, the DL technique is attracting increasing interest (38). The results of 10-fold CV analysis displayed performance consistency, indicating the reliability and robustness of the model (**Table 1**). This autoencoder framework was more efficient at identifying features related to relapse compared with others, with a C-index of 0.767. Li et al. (13) used the DL method combined with another computational method, namely similarity network fusion (SNF), for prediction of PCa relapse. Univariate Cox regression analysis, K-means clustering algorithms for the autoencoder model, and spectral clustering algorithms for SNF were then performed sequentially. Finally, six significantly overlapping biomarkers were considered. These valuable biomarkers could contribute to the early detection of high-risk relapse patients. However, limited omics data and only six biomarkers had a C-index value of 0.713, and an external validation set was not included. A most recent study identified relapse-related genes using core enrichment genes extracted from KEGG pathways *via* GSEA for univariate Cox regression analysis. The model was constructed using the Lasso method and a KM plot was mapped. The receiver operating characteristic (ROC) curve of the model was then used to evaluate predictive efficiency. The results showed that the area under the curve (AUC) for 3- and 5-year time-dependent ROC curves were 0.739 and 0.729 (39). Of equal importance, our model was also validated using five additional validation sets, each associated with a different omics level (mRNA, miRNA, DNA methylation, CNVs and lncRNA). The Lasso model constructed with these molecular labels according to model_3 was equally well able to classify these external validation sets into high relapse-risk and low relapse-risk subgroups (**Figure 2**), indicating that the relapse-related subgroups clustered using the DL-based model_3 have a broad spectrum of biological significance.

Using this model, more than 1000 differential genes were identified between S1 and S2 subgroups. VWA5B1, UGT2B15, and UTS2B were significantly up-regulated. In a previous study, von Willebrand factor (vWF) antigen was differentially abundant between patients with PCa or BPH and other prostatic diseases (40). Of note, the level of vWF antigen was elevated in patients with metastases, compared to localized PCa. However, VWA5B1 has not been reported in PCa, which may be a potential biomarker or target, but confirming this requires further research. The other two DEGs are closely related to PCa. In particular, UGT2B15 contributes to PCa risk, diagnosis and disease progression (41, 42), and 3α-diol-17 glucuronide, a product of UGT2B15/B17, is linked to prostate volume changes, indicating that this metabolite might serve as a biomarker of androgen activity. Moreover, UGT2B15 is one of the main determinants controlling the expression of target genes of androgen receptors in PCa cells (43). In addition, the UGT2B15 Asp85Tyr polymorphism is associated with PCa risk (41). Urotensin II receptor (UT) is involved in regulating the biological functions of urotensin II and UTS2B in mammals (44). UT mRNA expression was decreased in androgen-independent DU145 and PC3 cells, but increased in androgen-dependent LNCaP cells, and UT expression was strongly correlated with the prognosis of PCa, providing a potential prognostic marker for this disease (45). In summary, these three significantly up-regulated genes were directly or indirectly correlated with the progression and prognosis of PCa. All the identified DEGs may assist the prediction or prevention of PCa relapse.

Various up- and down-regulated genes were respectively enriched in GO and KEGG terms. Organelle fission enrichment has been linked to tumor tissues (46, 47). Hec1, a component of the nuclear division cycle 80 complex, was found to be elevated and associated with cancer progression in PCa (48). Emerging evidence suggests that chromosome segregation plays a vital role in PCa tumorigenesis, development and bone metastasis (49, 50). Neuroactive ligand-receptor interactions contribute to therapy-related neuroendocrine PCa, a lethal castration-resistant PCa subtype (51, 52). Both the initiation and progression of PCa have been associated with enhanced cell proliferation and cell cycle dysregulation (53).

GO/KEGG analyses focus on differential genes, and target a subset of genes that are significantly different between groups, and may therefore miss genes that are not significantly different but biologically significant. By contrast, GSEA identifies a set of genes with concordant differences from an expression matrix of all genes, and therefore takes into account genes that are less different. We applied GSVA, and the results suggested several relapse-related hallmarks/pathways of PCa, including 5 up-regulated and 13 down-regulated in the S2 subgroup. Several studies have proved that E2F factors play a critical role in mediating cell cycle gene expression and progression in PCa (54, 55). Nucleolar and spindle-associated protein (NuSAP), which binds DNA to the mitotic spindle, is associated with relapse after RP, and its promoter region contains two CCAAT motifs and binding sites for E2F, overexpression of which appears to be mediated partly by E2F1 activation (56). Similarly, the G2/M checkpoint plays a vital role in the cell cycle. Caspase-8, depletion of which can result in G2/M arrest, is involved in DNA damage (57). Furthermore, MYC enhances the expression of related genes to help cancer cells survive, grow, proliferate and metabolize, and MYC plays a central role in PCa according to tissue proteomics research (58). In summary, our results reconfirmed numerous genes or pathways closely linked with PCa, identified some potential tumor markers or therapeutic targets for PCa relapse, and revealed that DNA damage repair-related pathways, such as nuclear division and chromosome segregation, were significantly enriched in the S2 subgroup.

We investigated CNVs and found that 30.18% of genes had a significantly higher proportion of CNVs in the high relapse-risk S2 subgroup. As we all know, CNVs may be associated with malignancy through the accumulation of driver aberrations, and genomic instability can increase because DNA damage responses are absent and replication pressure is elevated in cancer cells (59, 60). Interestingly, cancer cells might be more vulnerable due to the relative specificity of these defects, which also has potential for increasing therapeutic indices of antineoplastic therapies, thereby improving the prognosis of cancer patients. Several clinical studies assessed the safety and effectiveness of state-of-the-art strategies such as DNA repair-targeted agents in various cancers (61, 62). Higher genomic instability was suggested for metastatic PCa (mPCa), based on the observation that the burden of CNVs and the weighted genome instability index were significantly higher in mPCa than localised PCa (63). Moreover, a significant correlation was observed between the burden of CNVs and PCa relapse and death (64, 65), indicating the potential of CNVs as prognostic biomarkers (66), consistent with our results.

Importantly, GO enrichment analysis of up-regulated DEGs (**Figure 4A**) yielded consistent results, suggesting a strong correlation between genomic or chromosomal instability and PCa relapse. Massive alterations in genetic information are the main feature distinguishing cancer cells and healthy cells. In addition to point mutations and small insertions/deletions (indels), large-scale changes occur, including chromosomal rearrangements, and chromosome gains and losses (individual or entire sets) (67). In other words, chromosome mis-segregation is rare in normal tissues, but chromosome (whole or part) gains and losses are common in cancer tissues. This chromosomal instability is correlated with intra-tumor heterogeneity, and it contributes to resistance to medical therapy as well as adverse outcomes of disease (68). Analysis of the distribution of these CNVs in chromosomes has important implications for future clinical and research studies on PCa relapse. Our CNV analysis results revealed that the S2 subgroup expressed significantly more genes on chromosomes 7 and 8 than did the S1 subgroup. Copy number-induced alterations are considered to be critical for tumor evolution (67). A 1991 study suggested that centromeric CNVs of chromosome 7 are closely related to tumor histological grade, and might be highly predictive for tumor aggressiveness in human bladder cancer (68). Alcaraz et al. (69) reported that aneuploidy and aneusomy of chromosome 7 are generally observed in the poor prognosis PCa patients. Meanwhile, allelic loss is frequently observed on the short arm of chromosome 8 (70). Ichikawa et al. (71) applied the microcell-mediated chromosome transfer technique to introduce human chromosome 8 into highly metastatic rat PCa cells and found that metastatic ability was suppressed, but similar trends were not observed in growth rate or tumorigenicity, indicating that chromosome 8 contains genes inhibiting metastasis of PCa, and implying a vital role in the progression of PCa. Regardless, chromosomes 7 and 8 may be correlated with PCa relapse according to current and previous research.

Interestingly, we also observed that immune infiltration might be involved in PCa relapse from the functional enrichment analysis of multiple differential CNVs (**Figure 8D**) and DEGs (**Figure 4D**). Emerging evidence shows that tumor immune cell infiltration, a significant hallmark of the tumor microenvironment, is an enormous contributing factor to therapeutic circumvention, cancer progression, and subsequent adverse outcomes (72). We calculated the proportion of infiltrating immune cell subsets of the two subgroups using the CIBERSORT algorithm (https://cibersort.stanford.edu/) and identified four TIICs with a significant difference.

However, four types of TIICs were not all elevated in the high relapse-risk S2 subgroup. Among them, monocytes were more abundant in the low relapse-risk S1 subgroup (**Figure 9D**). In a clinical study on 1107 participants, histopathological findings showed that patients with positive prostate biopsy had higher monocyte counts than negative patients, and multivariate Cox regression analyses showed that a high monocyte count was an independent prognostic factor of both cancer-specific and other mortalities (73). Even when adjusted by clinicopathological signatures, these outcomes were statistically significant, further confirming the independent correlations between high monocyte count and poor prognosis of PCa, contrary to our PCa relapse results. Therefore, whether monocytes differ during distinct stages of PCa, and whether this plays a major regulatory role in PCa progression, requires further investigation. Further studies of the influence of monocytes on PCa are also warranted. Recent work on aging men undergoing RP showed that the peripheral monocyte count was not linked to long-term results in PCa (74). The study included black and white RP men, but the peripheral monocyte count was not found to be a useful marker of PCa long-term results. One limitation of this work was the lack of Asian participants. Macrophages are important in cancer, too. Macrophages, derived from circulating monocytes, can be routinely classified into M1-type and M2-type macrophages. Classically activated M1 macrophages, acting as part of the innate immune response, are of great significance in the fight against invading pathogens, while activated M2 macrophages, also known as M2 tumor-associated macrophages, are vital components of tissue restoration and tumor promotion (75). M2 macrophages are involved in tumor development in various ways. They can directly interact with T cells and secrete factors associated with immunosuppression to inhibit CD8+ T cell immunity against cancer (72). Furthermore, some M2 macrophages influence tumor development *via* the destruction of antitumor T cell immunity, providing novel perspectives for immune tolerance and escape properties of cancers, consistent with our results.

Despite our best efforts, there remain some limitations with the current work. Firstly, because some raw data cluster labels were absent and survival information was lacking, it was difficult to compare directly with previous research. To confirm its predictive performance and direct application value in clinical practice, five external validation sets consisting of different omics data were applied, and the results were encouraging. Secondly, it remains unclear how many of the novel biological features we identified are linked to PCa relapse. Despite this, the results provide new directions for further exploration. Thirdly, while we demonstrated the effectiveness and robustness of this DL model in various ways, future experiments and clinical data are needed to realize its potential.

In summary, we successfully constructed a DL-based predictive model integrating five-omics for PCa relapse with a significant relapse difference between two subgroups. Furthermore, validation using five independent omics datasets confirmed its robustness. A number of critical DEGs, pathways, and functions were found to be associated with PCa relapse. The model provides new insights for distinguishing relapse-risk patients, and it could benefit patients due to its early predictive ability and subsequent early therapeutic intervention. The findings contribute to our current understanding of PCa relapse, and the developed model may serve clinical applications and support decision-making.

## Data Availability Statement

Publicly available datasets were analyzed in this study. This data can be found here: The 417 PCa samples were obtained from the The Cancer Genome Atlas (TCGA; https://www.cancer.gov/). The additional validation sets (GSE70768, GSE26367, GSE26126, and GSE21035) were collected from Gene Expression Omnibus (GEO; http://www.ncbi.nlm.nih.gov/geo/). Hallmark gene sets c2.cp.kegg.v6.2.symbols.gmt, c2.cgn.v6.2.symbols.gmt, c5.all.v6.2.symbols.gmt and c6.all.v6.2.symbols.gmt were downloaded from the MSigDB molecular signatures database (http://software.broadinstitute.org/gsea/msigdb). Access codes for this article are listed in https://github.com/foolman1990/deep-learning-multiomics.

## Ethics Statement

This study did not need approvals by the Ethics Committee of Jinshan Hospital, Shanghai, China according to the regulations. This study fully complied guidelines of GEO and Array Express.

## Author Contributions

All authors contributed to the article and approved the submitted version. Study design and project administration: GC, and ZS; Data collection, analysis and interpretation: ZS, ZW, DH, CZ, SW, JL, and FC; Writing-review and editing of the manuscript: ZW, ZS, GC.

## Funding

This work was supported by grants from the Natural Science Foundation of Shanghai (NO. 18ZR1405800; NO. 22ZR1409700) and the Project for Key Medical Specialty Construction in Jinshan District (6th Period, Type A; No. JSZK2019A03) to GC.

## Conflict of Interest

The authors declare that the research was conducted in the absence of any commercial or financial relationships that could be construed as a potential conflict of interest.

## Publisher’s Note

All claims expressed in this article are solely those of the authors and do not necessarily represent those of their affiliated organizations, or those of the publisher, the editors and the reviewers. Any product that may be evaluated in this article, or claim that may be made by its manufacturer, is not guaranteed or endorsed by the publisher.
