# Supplementary material for: Deep Learning-Based Multi-Omics Integration Robustly Predicts Relapse in Prostate Cancer
Source: Front Oncol. 2022 Jun 23;12:893424. doi: 10.3389/fonc.2022.893424 (PMC9259796; doi:10.3389/fonc.2022.893424)
Supplement: Supplementary file 1 [file DataSheet_1.docx]

**
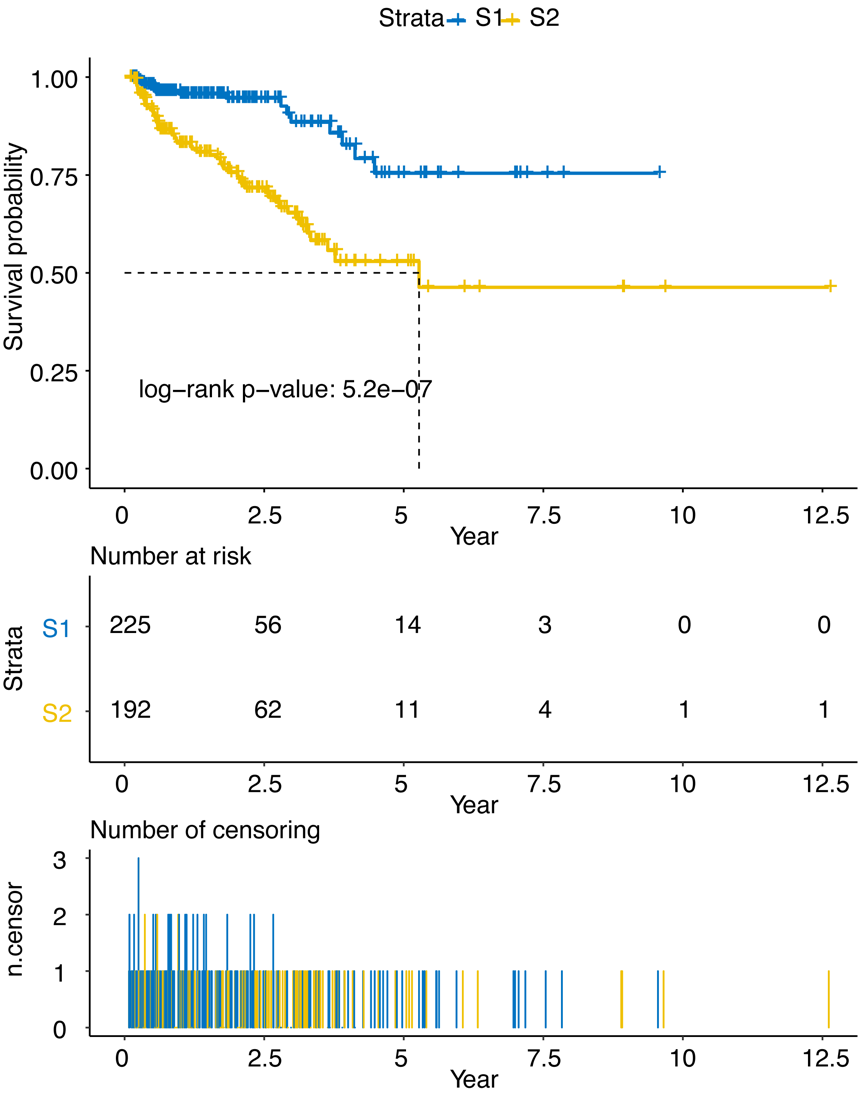
**

**Supplementary Figure 1 |** Significant survival differences for model_8. Relapse-related deep-features of model_8 were used for subgrouping, and the KM plot was used to show the difference in relapse levels between the two subgroups with a log-rank *P*-value = 5.3e-07 > 6e-09 and the time of half relapse is more than 5 years, both of which were worse than model_3.

**Supplementary Table 1 |** Lasso screening of relapse-related significant labels in TCGA mRNA, lncRNA, miRNA, methylation and CNV data according to the subgroups obtained from model_3, and then the Lasso model was constructed. The results are as follows.

| **mRNA, N = 43** | | | | | | | | |
| --- | --- | --- | --- | --- | --- | --- | --- | --- |
| TSSK3 | PRCC | FMOD | ITGB1BP1 | EIF5B | DLX1 | XYLB | ADPRH | MCCC2 |
| DOK3 | HIST1H2BO | WTAP | ZNF783 | ARMCX1 | MAGEA8 | ATP6V0D2 | PHYHD1 | BBS1 |
| CEP164 | NTF3 | BTBD11 | C12ORF76 | PGAM5 | ZNF605 | PCCA | CIDEB | CGRRF1 |
| LITAF | RPH3AL | SHBG | ANKRD13B | MMP28 | KRT14 | POLG2 | PGS1 | SOX12 |
| GPX3 | PPME1 | SSTR5 | UBE2V1 | KCNK6 | RINL | SRRM5 |  |  |
| **miRNA, N = 22** | | | | | | | | |
| hsa_let_7c | hsa_let_7d | hsa_mir_103a_2 | hsa_mir_126 | hsa_mir_133b | hsa_mir_135a_1 | hsa_mir_137 | hsa_mir_221 | hsa_mir_192 |
| hsa_mir_141 | hsa_mir_143 | hsa_mir_146b | hsa_mir_15b | hsa_mir_16_1 | hsa_mir_184 | hsa_mir_187 | hsa_mir_20b | hsa_mir_21 |
| hsa_mir_199a_1 | hsa_mir_210 | hsa_mir_205 | hsa_mir_212 |  |  |  |  |  |
| **lncRNA, N = 24** | | | | | | | | |
| SNHG3 | LINC01135 | AC245595.1 | PCBP1_AS1 | LINC01114 | PCGEM1 | AC073316.1 | AC018645.2 | LINC00158 |
| FIRRE | AP003469.2 | AC103706.1 | SNHG1 | AP000911.1 | AC004801.5 | SLC25A30_AS1 | AL137060.1 | LINC00205 |
| LINC01588 | LINC00640 | MEG9 | AC012236.1 | LINC02193 | AC015922.3 |  |  |  |
| **methylation genes, N = 30** | | | | | | | | |
| AMY2B | ARMCX2 | BCL9L | CBWD1 | CCL19 | FMO3 | FOXP2 | FUBP1 | GDAP1L1 |
| HOXA9 | IL13RA2 | IMP4 | PEX10 | PI15 | PYHIN1 | RECQL4 | RHOBTB3 | RIN1 |
| SPATA8 | STAT6 | TMED2 | TMPRSS11B | TOMM20 | TSPYL2 | USH2A | ZBTB7B | ZMYM5 |
| HERPUD1 | SLC35A3 | ZMYND10 |  |  |  |  |  |  |
| **CNV genes, N = 72** | | | | | | | | |
| ABCC5 | ACAT2 | ADAM28 | ADAM7 | ADAMDEC1 | ADRA1A | ADRA2C | AGTRAP | ANKMY1 |
| ATP12A | AURKB | C12ORF4 | C12ORF54 | CAB39L | CCDC82 | CCDC87 | CCS | CCT3 |
| CHD7 | CILP | CLK2 | EFNA5 | EWSR1 | FSTL1 | FXR2 | GCOM1 | GTF2E1 |
| MAD2L2 | MGRN1 | MINPP1 | MRPL18 | MRPS14 | MTMR6 | NKX3_1 | NPR1 | OR8S1 |
| PBK | PNLDC1 | PPFIBP1 | PRPF40B | PTPN12 | RABL3 | RAD51AP1 | RBM14 | RGR |
| RNF122 | RNF17 | S100A13 | S100A14 | S100A16 | SLC26A9 | SLC35D2 | SLC41A3 | SLCO6A1 |
| TCP1 | TG | THBS3 | TLX1 | WTAP | ZBTB20 | ANKRD11 | CD200R1 | HABP4 |
| PAQR6 | RGS18 | SMG5 | TCF3 | RHBDD3 | PARP16 | ILF2 | CENPJ | AP2M1 |
